# Working Memory Content Is Distorted by Its Use in Perceptual Comparisons

**DOI:** 10.1177/09567976211055375

**Published:** 2022-04-22

**Authors:** Keisuke Fukuda, April E. Pereira, Joseph M. Saito, Ty Y. Tang, Hiroyuki Tsubomi, Gi-Yeul Bae

**Affiliations:** 1 Department of Psychology, University of Toronto; 2 Department of Psychology, University of Toronto Mississauga; 3 Department of Psychology, University of Waterloo; 4 Department of Psychology, Arizona State University; 5 Department of Humanities, University of Toyama

**Keywords:** working memory, memory distortion, individual differences, open data

## Abstract

Visual information around us is rarely static. To perform a task in such a dynamic environment, we often have to compare current visual input with our working memory (WM) representation of the immediate past. However, little is known about what happens to a WM representation when it is compared with perceptual input. To test this, we asked young adults (*N* = 170 total in three experiments) to compare a new visual input with a WM representation prior to reporting the WM representation. We found that the perceptual comparison biased the WM report, especially when the input was subjectively similar to the WM representation. Furthermore, using computational modeling and individual-differences analyses, we found that this similarity-induced memory bias was driven by representational integration, rather than incidental confusion, between the WM representation and subjectively similar input. Together, our findings highlight a novel source of WM distortion and suggest a general mechanism that determines how WM interacts with new visual input.

**Statement of Relevance**

Imagine a person who witnesses a hit-and-run traffic accident. When the witness tries to identify the license plate number of the car involved, a bus occludes their sight of the car. When the bus passes, they need to find the car by comparing the cars they see now with the memory of the car that committed the accident. In doing so, they might assume that their memory of the car remains intact as it is compared with the cars they see now. However, the present study shows that this assumption is simply not valid: As a memory representation of the immediate past (i.e., working memory) is compared with new perceptual information, the memory report becomes systematically biased toward perceptually similar input. Thus, our findings reveal an overlooked vulnerability in working memory and suggest that the witness may misreport the license plate of a similar, but different, car to the police.

Many daily activities rely on our ability to maintain accurate mental representations of the immediate past to guide our behavior. For example, consider a Good Samaritan who witnesses a phone falling out of a passerby’s pocket on a busy street. The Good Samaritan would look down to pick up the phone in hopes of returning it to its owner. After doing so, they would need to find the owner again by comparing their memory of the owner’s appearance with the other people they see on the street. Past studies have demonstrated that working memory (WM) can actively maintain a small amount of task-relevant information (e.g., the owner’s appearance) over a brief delay when the information is not perceptually present (e.g., while looking down on the street to pick up the phone; Luck & Vogel, 2013; Ma et al., 2014). However, little is known as to whether WM remains intact as it is used for memory-guided perceptual comparisons.

Although one might assume that WM remains intact as it is used, past studies have shown that WM can be interfered with by subsequent perceptual inputs introduced during maintenance (e.g., Bennett & Cortese, 1996; Magnussen & Greenlee, 1992; Nemes et al., 2012; Sun et al., 2017; Teng & Kravitz, 2019) and at the time of memory reporting (Makovski et al., 2008; Wang et al., 2018). For example, in a previous study, participants were asked to maintain a color in WM while completing a rapid serial visual presentation (RSVP) task in which a stream of letters was presented on a differing color patch (Teng & Kravitz, 2019). The researchers found that the color representation maintained in WM was distorted by the task-irrelevant color patch, especially when the two colors were physically similar to each other. Therefore, WM seems vulnerable to perceptual interference.

However, the true nature of this interference remains unknown. For example, it is unclear whether the memory distortion occurs if a WM representation remains within the focus of internal attention. Given that the intervening RSVP task used by Teng and Kravitz (2019) drew attention away from WM maintenance, the memory distortion may be contingent on disruption of active WM maintenance (Makovski et al., 2008; Wang et al., 2018). It is also possible that the WM representation was not necessarily distorted but was occasionally replaced by the intervening stimulus instead (i.e., swap errors), making WM reports appear biased (Bays et al., 2009). Lastly, because previous studies did not measure the perceived similarity between WM and intervening stimuli, it is not known whether the interference was dictated by the physical similarity or perceived similarity between them.

We addressed these questions in the present study by having participants remember a single visual feature (i.e., color or shape) in WM and compare it with new stimuli. Therefore, the WM had to be actively maintained so that it could be compared with new stimuli. In three experiments, we found that WM reports were distorted, especially when participants perceived the new stimuli as similar to the WM. Critically, we found that this similarity-induced memory bias was larger following perceived similarity than perceived dissimilarity of new stimuli, even after controlling for their physical similarity. We further validated the systematic effect of subjective similarity by demonstrating that memory biases induced by an initial similarity judgment were “canceled out” by an additional judgment that biased the WM in an opposing direction. Computational modeling revealed that the similarity-induced memory bias was better accounted for by representational integration between WM and perceptually similar input than by probabilistic swap errors. Thus, our findings reveal an overlooked window of vulnerability in WM and suggest a general mechanism that determines how a new perceptual input interacts with WM (e.g., Kiyonaga et al., 2017; Rademaker et al., 2019; Sun et al., 2017).

## Experiments 1a and 1b

**Fig. 1.**
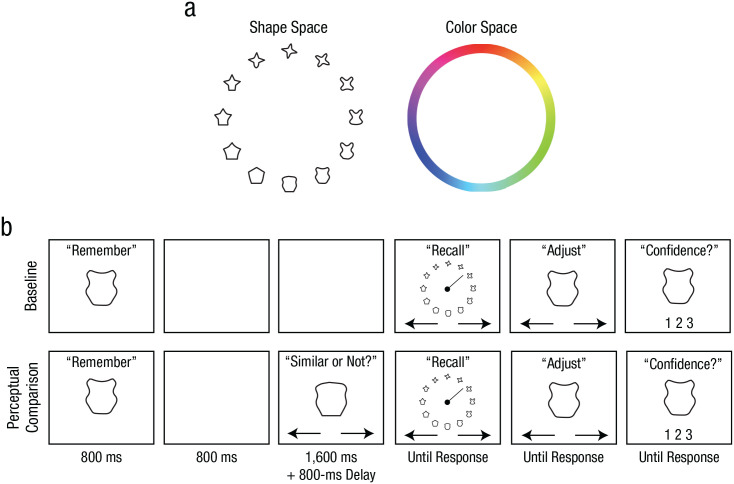
Stimulus spaces and example trial procedures from Experiments 1a and 1b. The stimulus spaces used for Experiments 1a (shape task) and 1b (color task) are shown in (a). Trial procedures from the shape task (b) are shown separately for the baseline and perceptual-comparison conditions. In each condition, participants were first presented with a target shape that they attempted to remember across a brief maintenance interval. Following the maintenance interval, participants reported the target shape by rotating a line indicator to select from a continuous wheel. Participants then adjusted their selection, if desired, to best match their memory before submitting their report with a confidence rating. In the perceptual-comparison condition, participants also performed a perceptual comparison on a probe shape that was presented during the maintenance interval by indicating whether the probe shape was similar or dissimilar to the target shape. The color task was identical to the shape task except for the stimulus type and the stimulus wheel.

To examine the consequences of comparing WM with perceptual inputs, we had participants remember a simple stimulus (i.e., shape in Experiment 1a and color in Experiment 1b) and compare it with a new perceptual input (Fig. 1). Subsequently, participants reported their memory of the original stimulus as precisely as possible. Here, we predicted that the WM report would be biased toward the new input, especially when the new input was subjectively similar to the WM.

### Method

#### Participants

Related experiments examining the memory bias for orientation memory (Rademaker et al., 2015) reported large effect sizes (Cohen’s *d* > 1.5). Because we used different stimuli, we anticipated a medium to large effect size (Cohen’s *d* = 0.8). A power analysis (conducted in G*Power Version 3.1; Faul et al., 2007) determined that at least 15 samples would be necessary to detect such an effect with a statistical power of .8. We recruited 16 participants (11 female; mean age = 23.2 years) for Experiment 1a and 16 participants (10 female; mean age = 22.6 years) for Experiment 1b. Participants provided informed consent, and the study was approved by the University of Toronto Research Ethics Board.

#### Apparatus, stimuli, and procedure

Stimuli were generated in MATLAB (The MathWorks, Natick, MA) using the Psychophysics Toolbox (Brainard, 1997) and were presented at 60 Hz on an LCD monitor. Viewing distance was approximately 60 cm. The shape stimulus set used in Experiment 1a contained 360 shapes whose circular continuity was empirically validated (Li et al., 2020). The color stimuli used in Experiment 1b comprised 360 equally spaced color values sampled from Commission Internationale de l’Éclairage (CIE) L*a*b* space with a* centered at 20 and b* centered at 38 with a radius of 60. L* was set to 70.

Participants completed multiple trials in the baseline and perceptual-comparison conditions (Fig. 1b). In the baseline condition, participants were presented with one stimulus—either a shape (3.8° × 3.8°) or a colored circle (5.2° diameter) for 800 ms. After a 3,200-ms retention interval, participants were presented with a circular stimulus wheel (15.4° diameter). For the shape task, the stimulus wheel was composed of 18 equidistant shapes (20° apart in the shape space). For the color task, it was a color wheel composed of the 360 colors (1° per color). To report the remembered stimulus, they first selected an item that best matched the original stimulus from the stimulus wheel by rotating a line indicator using the left and right arrow keys on the keyboard. After the indicator pointed toward the desired item, participants pressed the space bar to confirm the selection. The selected item then appeared at the center of the screen for optional refinements using the left and right arrow keys. When satisfied, participants indicated their confidence in their report (1 = high confidence, 2 = low confidence, 3 = no confidence) by pressing one of three keys on the keyboard. No time limit was imposed on the memory report in order to emphasize accuracy.

In the perceptual-comparison condition, a probe stimulus was presented 800 ms after the offset of the original stimulus, and participants indicated whether it looked similar or dissimilar to the original stimulus by pressing a key. The probe stayed on the screen for 1,600 ms. The probe was randomly sampled from 16° to 105° away from the original stimulus on each trial. After an 800-ms delay following the probe presentation, participants reported the original memory item as in the baseline condition. Participants performed 12 experimental blocks, and each block contained 12 baseline and 36 perceptual-comparison trials.

#### Analysis

For each trial, a signed response offset was calculated in relation to the probe. A positive sign indicated a response offset toward the probe (for the distribution of raw response offsets, see the supplementary material available at https://osf.io/79kbm/). For the baseline trials, the sign of the response offset was randomly assigned. To quantify the magnitude of the bias, we computed the mean of the signed response offsets for each condition. We focused on trials with high-confidence memory reports (> 68% of trials, or > 83 trials, in all conditions; for the same pattern of results with all trials included, see the supplementary material available at https://osf.io/79kbm/).

To isolate the effect of perceived similarity on the memory bias, we first identified the probe distances that resulted in both similar and dissimilar judgments on separate trials within subjects (*ambivalent probe distances*). For each ambivalent probe distance, we calculated the mean bias magnitude following similar and dissimilar judgments separately. The mean bias magnitudes following similar and dissimilar judgments were then averaged across all ambivalent probe distances. This procedure isolated the effect of perceived probe similarity (indicated by participants’ judgments) on the bias magnitude while equating the effect of physical probe similarity (determined by the sampling procedure).

### Results

**Fig. 2.**
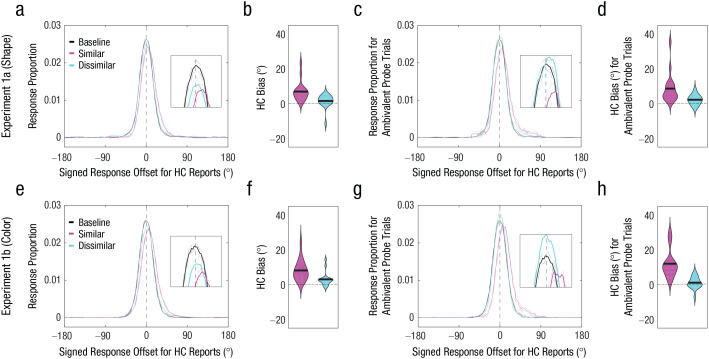
Results of Experiments 1a (top row) and 1b (bottom row). Probability distributions of signed response offsets for high-confidence (HC) reports (a, e) are shown for the baseline condition and for similar and dissimilar probe judgments in the perceptual-comparison condition. For demonstration purposes, the response proportion for a given signed response offset was computed by calculating the mean response proportion across a 30° window centered around the signed response offset. Positive offsets indicate memory bias toward the first similar probe. The broken lines indicate within-subjects standard errors of the mean. Bias magnitude for HC memory reports (b, f) is shown for similar (pink) and dissimilar (blue) probe judgments. In each violin plot, the thick horizontal line indicates the mean across participants, and the width of the violin indicates the density of the data. Positive values indicate memory bias toward the probe. Probability distributions of signed response offsets for HC reports for ambivalent probe trials (c, g) are shown for the baseline condition and for similar and dissimilar probe judgments in the perceptual-comparison condition. Bias magnitude for HC memory reports for ambivalent probe trials (d, h) is shown for similar (pink) and dissimilar (blue) probe judgments. In each violin plot, the thick horizontal line indicates the mean across participants, and the width of the violin indicates the density of the data. Positive values indicate memory bias toward the probe.

Both shape (Experiment 1a) and color (Experiment 1b) memory reports exhibited an attraction bias (> 0°) toward the probe when the probe was judged to be similar to the memory item—shape: *M* = 7.61°, *t*(15) = 4.70, *p* < .001, 95% confidence interval (CI) = [4.28°, 10.94°], Cohen’s *d* = 1.18 (Figs. 2a and 2b); color: *M* = 8.14°, *t*(15) = 5.47, *p* < .001, 95% CI = [4.97°, 11.32°], Cohen’s *d* = 1.37 (Figs. 2e and 2f). We also found some evidence for an attraction bias when the probe was judged to be dissimilar to the original memory item—shape: *M* = 1.49°, *t*(15) = 1.48, *p* = .159, 95% CI = [–0.65°, 3.64°], Cohen’s *d* = 0.37; color: *M* = 3.03°, *t*(15) = 2.93, *p* = .01, 95% CI = [0.83°, 5.12°], Cohen’s *d* = 0.73—but the bias magnitude was significantly smaller than when the probe was judged to be similar—similar shape versus dissimilar shape: *M* = 5.42°, *t*(15) = 3.14, *p* = .007, 95% CI = [1.74°, 9.10°], Cohen’s *d* = 0.78; similar color versus dissimilar color: *M* = 5.11°, *t*(15) = 2.98, *p* = .009, 95% CI = [1.45°, 8.77°], Cohen’s *d* = 0.74. The trials with ambivalent probe distances (shape task: mean range of probe distance = 20.1°–97.7°; color task: mean range of probe distance = 19.3°–97.6°) produced larger attraction biases when the probes were perceived to be similar rather than dissimilar to the original memory items—shape task: Δ*M* = 6.19°, *t*(15) = 3.01, *p* = .009, 95% CI = [1.81°, 10.58°], Cohen’s *d* = 0.75 (Figs. 2c and 2d); color task: Δ*M* = 10.92°, *t*(15) = 4.50, *p* < .001, 95% CI = [5.75°, 16.08°], Cohen’s *d* = 1.13 (Figs. 2g and 2h).

## Experiment 2

**Fig. 3.**
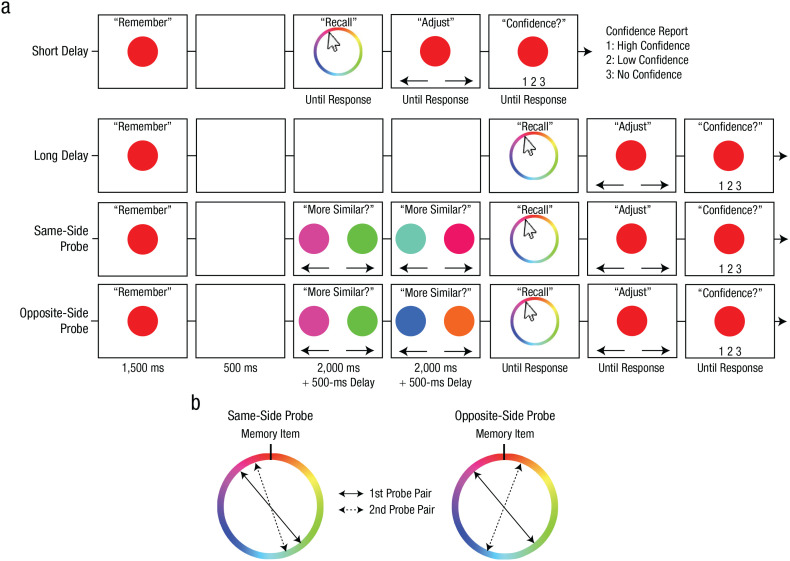
Trial procedures and probe-sampling procedures for Experiment 2. Trial procedures from the color task (a) are shown separately for the baseline (short delay, long delay) and experimental (same-side probe, opposite-side probe) conditions. In each condition, participants were first presented with a target color that they attempted to remember across a brief maintenance interval. Following the maintenance interval, participants reported the target color by clicking on a continuous wheel. Participants then adjusted their selection, if desired, to best match their memory before submitting their report with a confidence rating. In the experimental conditions, participants also performed two consecutive perceptual comparisons during the maintenance interval. In each comparison, participants selected which of two simultaneously presented probe colors was more similar to the target color. The sampling of the similar probes is depicted in (b). In the same-side-probe condition, the similar probes were both sampled from the same side of the circular color space relative to the target. In the opposite-side-probe condition, the similar probes were sampled from opposite sides of the target. The dissimilar probes were sampled 180° from the similar probes in both pairs. The shape task was identical to the color task except for the stimulus type and the stimulus wheel.

Experiment 1 demonstrated that WM reports were biased when WM was directly compared with a subjectively similar probe. In Experiment 2, we sought to replicate this similarity-induced memory bias in a modified paradigm. More precisely, participants clicked a mouse to report WM and performed two-alternative forced-choice judgments for perceptual comparisons (Fig. 3). More importantly, considering that many behaviors rely on the maintenance of unbiased WM representations, we examined whether the similarity-induced memory bias can be corrected by biasing WM in the opposite direction in a subsequent perceptual judgment. If the bias can be corrected by subsequent perceptual judgments, then its magnitude should be smaller when the similar probes in two consecutive judgments were sampled from opposing directions (i.e., clockwise and counterclockwise) than from the same direction.

### Method

#### Participants

On the basis of the results of Experiment 1 (Cohen’s *d* > 0.73), we anticipated a medium to large effect size (Cohen’s *d* = 0.73). A power analysis (conducted in G*Power Version 3.1; Faul et al., 2007) determined that 22 samples were necessary to detect such an effect with a statistical power of .9. After providing written informed consent to protocols approved by the University of Toronto Research Ethics Board, 32 individuals (24 female; mean age = 19.3 years) participated. The data from four participants were removed because they did not complete the experiment (*n* = 1), failed to follow instructions (*n* = 1), or too infrequently made high-confidence memory reports (i.e., < 15% for the baseline conditions; *n* = 2). As a result, 28 participants’ data were analyzed.

#### Procedure

The procedure was similar to that of Experiment 1. Participants performed four blocks of the color task and four blocks of the shape task in a pseudorandomized order. In the baseline conditions (Fig. 3a), participants remembered the original stimulus over a 500-ms (short delay) or a 5,500-ms (long delay) interval. Two delays were introduced to establish the baseline response-offset distributions for the perceptual-comparison conditions (for an example of the effect of delay on WM precision, see Rademaker et al., 2018). After the delay, participants used a mouse to click on an item that best matched the original stimulus, fine-tuned their response using key presses, and then reported their confidence as in Experiment 1.

In the perceptual-comparison conditions, participants performed two intervening perceptual comparisons 500 ms after the original stimulus offset. Because subjective similarity of a given perceptual probe varied across trials, we controlled the subjective similarity of a physically similar probe (e.g., 30° away from the target) by presenting it together with a physically dissimilar probe (180° away from the similar probe) and had participants identify the more similar probe from the pair (Fig. 3). In each comparison, two probes were presented on each side of the screen (5.2° from the center of the screen), and they reported which probe looked more similar to the original stimulus by pressing either the left or right arrow key. One of the probes was randomly sampled from ±16° to 45° away from the original stimulus (i.e., similar probe). The other probe was sampled 180° away from the similar probe (i.e., dissimilar probe). The two probes remained on the screen for 2,000 ms regardless of the report. After the offset of the first pair of probes, a 500-ms delay followed, and the second pair of probes was presented for another similarity judgment (2,000 ms). After another 500-ms delay, participants reported the original stimulus in the same manner as the baseline conditions.

The two perceptual-comparison conditions differed in how the similar probes were sampled (Fig. 3b). In the same-side-probe condition, similar probes in each pair were sampled from the same side of the stimulus space relative to the memory item. In the opposite-side-probe condition, similar probes in each pair were sampled from opposite sides of the stimulus space relative to the memory item. Participants completed 40 trials for each condition in a pseudorandomized order.

#### Analysis

For each trial, a signed response offset was calculated as the response offset in the direction of the similar probe in the first probe pair (for the distribution of raw response offsets, see the supplementary material available at https://osf.io/79kbm/). Thus, a positive value indicates a response offset toward the first similar probe. For the baseline trials, the sign was randomly assigned. The magnitude of the memory bias was quantified as the mean of the signed response offsets for each condition. We focused on trials with high-confidence memory reports (> 58% of trials, or > 25 trials, in all conditions; for the same pattern of results with all trials included, see the supplementary material available at https://osf.io/79kbm/).

### Results

**Fig. 4.**
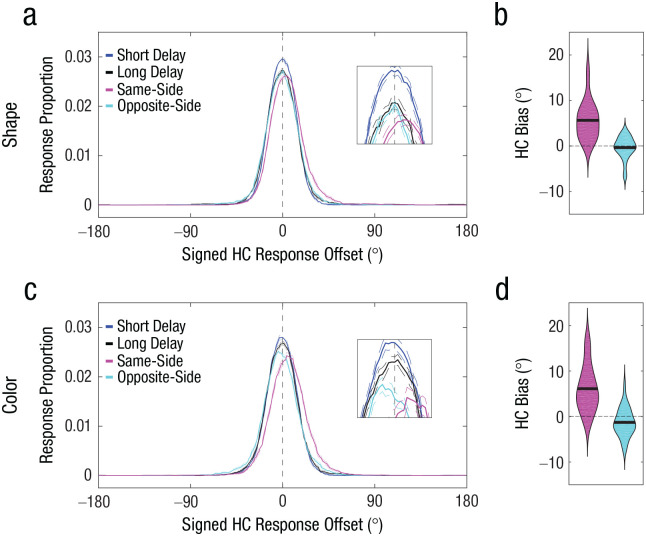
Results of the shape task (top row) and color task (bottom row) in Experiment 2. Probability distributions of signed response offsets for high-confidence (HC) reports (a, c) are shown for each of the four conditions in the shape task. For demonstration purposes, the response proportion for a given signed response offset was computed by calculating the mean response proportion across a 30° window centered around the signed response offset. Positive offsets indicate memory bias toward the first similar probe. The broken lines indicate within-subjects standard errors of the mean. Bias magnitude for HC memory reports in the shape task (b, d) is shown for same-side (pink) and opposite-side (blue) probe judgments. In each violin plot, the thick horizontal line indicates the mean across participants, and the width of the violin indicates the density of the data. Positive values indicate memory bias toward the first similar probe.

Accuracy in the perceptual-comparison conditions was near ceiling (shape task: *M* = 0.94, *SD* = 0.05 for the same-side probe, *M* = 0.89, *SD* = 0.07 for the opposite-side probe; color task: *M* = 0.95, *SD* = 0.04 for the same-side probe, *M* = 0.92, *SD* = 0.04 for the opposite-side probe), thus confirming that subjective similarity of the probe was successfully controlled. As can be seen from Figures 4a and 4c, the same-side-probe condition and the opposite-side-probe condition exhibited differential signed response offsets. The mean signed response offset for the same-side-probe condition exhibited a significant positive signed offset—shape task: *M* = 5.64°, *t*(27) = 7.17, *p* < .001, 95% CI = [4.02°, 7.25°], Cohen’s *d* = 1.36 (Fig. 4b); color task: *M* = 6.11°, *t*(27) = 6.30, *p* < .001, 95% CI = [4.12°, 8.09°], Cohen’s *d* = 1.19 (Fig. 4d)—indicating that the memory reports were attracted toward the similar probes. In contrast, the mean signed response offset for the opposite-side-probe condition exhibited a nonsignificant negative signed offset for shape, *M* = −0.36°, *t*(27) = −0.78, *p* = .441, 95% CI = [–1.32°, 0.59°], Cohen’s *d* = −0.15, and a small but significant negative signed offset for color, *M* = −1.29°, *t*(27) = 2.20, *p* = .037, 95% CI = [–2.49°, –0.09°], Cohen’s *d* = −0.42, indicating that the memory reports were, if anything, biased away from the first similar probe. The magnitude of the bias (i.e., absolute values) for the same-side-probe condition was statistically greater than the magnitude of the bias for the opposite-side-probe conditions—shape task: Δ*M* = 5.27°, *t*(27) = 7.46, *p* < .001, 95% CI = [3.82°, 6.72°], Cohen’s *d* = 1.41; color task: Δ*M* = 4.82°, *t*(27) = 3.88, *p* < .001, 95% CI = [2.27°, 7.37°], Cohen’s *d* = 0.73.

## Modeling of the Similarity-Induced Memory Bias

**Fig. 5.**
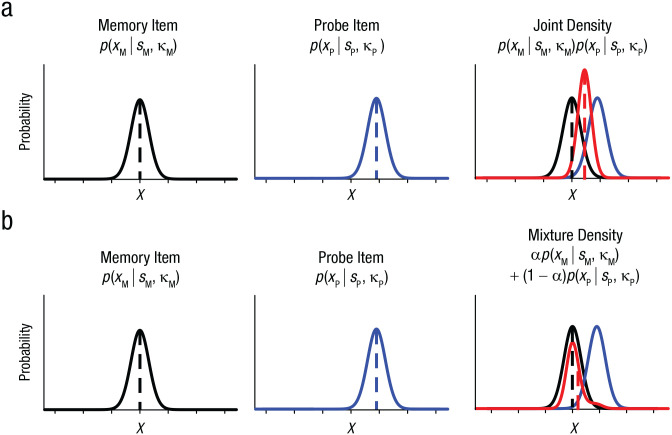
Two competing models of similarity-induced memory bias. In the joint-density model (a), noisy representation of a working memory item (*X*_M_) is assumed to follow a von Mises distribution centered at the sample stimulus (*S*_M_, dashed black vertical line) with some precision (κ_M_, left). Noisy representation of a probe item is also assumed to follow a von Mises distribution centered at the probe stimulus (*S*_P_, dashed blue vertical line) with some precision (κ_P_, center). When observers decide that the memory item and the probe item are perceptually similar, then the two items are integrated to produce a joint distribution (shown in red, right). As a result, the mean of the joint distribution is biased toward the probe item, as indicated by the dashed red vertical line. In the mixture-density model (b), the initial memory representation and the probe representation are assumed to follow von Mises distributions as in the joint-density model. However, this model assumes that some proportion of the memory reports is based on the probe representation. This can be accounted for by the mixture parameter (a). The original memory representation in this model is not biased, but the mean of the mixture distribution can be shifted toward the probe item (dashed red vertical line). Note that the schematics for both models depict the corresponding representational consequences for one trial with a given probe distance. For a simulation of multiple trials with varying probe distances as tested in the actual experiments, see the supplementary material available at https://osf.io/79kbm/.

Experiment 2 not only replicated the similarity-induced memory bias in a modified paradigm but also demonstrated that it could be corrected by an additional similarity judgment. One important question remains regarding the computational mechanism underlying the similarity-induced memory bias. One possibility is that a WM is integrated with a probe if the probe is subjectively similar to the WM (Fig. 5a; for a similar conceptualization, see Dubé et al., 2014). This integration can be accomplished via the formation of a joint density of the two, resulting in a biased WM representation toward a similar probe (Bae et al., 2015). Alternatively, the two representations may be independently represented in WM, but participants may occasionally report the probe item instead of the memory item by mistake, especially when the probe is similar to the memory item (Fig. 5b). Importantly, this mixture density can also produce a shifted response distribution depending on the frequency of the mistake (see the supplementary material available at https://osf.io/79kbm/). We compared these competing models by fitting them to the data obtained in Experiment 1.

### Method

Detailed descriptions of each model can be found in the supplementary material (https://osf.io/79kbm/). Here, we provide a summary.

For both the joint-density model and the mixture-density model, we assumed that both the memory (*X*_M_) and the probe (*X*_P_) representations follow von Mises distributions (denoted by φ) centered at the stimulus value (*S*_M_, *S*_P_) with some precision (κ_M_, κ_P_):

(1) $$p\left(X_{M}\;|\;S_{M}\right)=\phi \left(X_{M}\;|\;S_{M},\;\kappa_{M}\right)$$

(2) $$p\left(X_{P}\;|\;S_{P}\right)=\phi \left(X_{P}\;|\;S_{P},\;\kappa_{P}\right)$$

When the probe item (Equation 2) is perceived to be similar to the memory item (Equation 1), the joint-density (JD) model integrates the two representations in the following manner:

(3) $$p\left(X_{\mathrm{JD}}\;|\;S_{M},{\;S}_{P}\right)=\frac{p(X_{M}\;|\;S_{M})p(X_{P}\;|\;S_{P})}{\sum^{\;}p(X_{M}\;|\;S_{M})p(X_{P}\;|\;S_{P})}$$

The joint-density model (Equation 3) has four parameters. The two parameters for the center of the memory and the probe distributions were set by the actual stimulus values (S_M_, S_P_). The precision parameter for the memory item (κ_M_) was obtained by fitting a standard WM model (Zhang & Luck, 2008) to response offsets in the baseline condition in Experiment 2. However, we fitted the precision for the probe item within the model (κ_P_). Thus, the joint-density model has only one free parameter.

We fitted the joint-density model to each trial and each participant separately for Experiments 1a and 1b data sets. We used only high-confidence memory reports to avoid contamination by guessing or lapses of attention. On a given trial for a given participant, we constructed a joint-density distribution using *S*_M_ and *S*_P_ for that trial and κ_M_ for the participant and fitted the model by finding a probe precision (κ_P_) that minimized the difference between the average human response error collapsed across all the trials (including all the probe distances) and the average simulated response errors across all the simulated responses.

The mixture-density model (Equation 4) combines the two distributions via a mixture parameter (a). Namely, this model assumes that the final memory reports are either memory-based or probe-based. The proportion of each is determined by the mixture parameter:

(4) $$p\left(X_{\mathrm{Mix}}\;|\;S_{M},S_{P}\right)=\alpha p\left(X_{M}\;|\;S_{M}\right)+\left(1-\alpha \right)p\left(X_{P}\;|\;S_{P}\right)$$

 

All the other aspects of this model were identical to the joint-density model except that this model has an additional free parameter (a). Alpha was set to vary between 0 (0% memory-based reports) and 1 (100% memory-based reports).

### Results

**Fig. 6.**
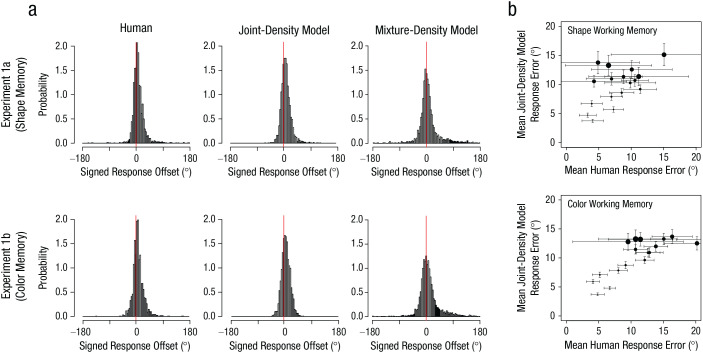
Observed and simulated memory bias in Experiments 1a (top row) and 1b (bottom row). Probability distributions of signed response errors (a) are shown separately for responses of human participants, predictions of the best-fitting joint-density model, and predictions of the best-fitting mixture-density model for each experiment. Correlations of bias magnitudes (b) between the simulated responses from the joint-density model and the observed responses from human participants in the shape and color tasks are shown for each of the 18 probe distances in each experiment. Positive values indicate a bias toward the probe item. The size of the dots represents the discretized physical distance of the probe (smaller dots = closer probes, larger dots = further probes). Vertical error bars indicate ±1 bootstrapped standard error of the simulated responses. Horizontal error bars indicate ±1 bootstrapped standard error of human data.

Figure 6a shows simulated response-offset distributions from the joint-density model and the mixture-density model along with observed human data (Experiments 1a and 1b). The peak of the simulated response distribution from the joint-density model was shifted positively, as in the human data. However, the distributions from the mixture-density model were positively skewed without this shift. This result suggests that the observed biases in the human data were more likely to be driven by representational integration than probabilistic confusion. Formal model comparisons using measurements of the sum of log-likelihood, Akaike information criterion, and Bayesian information criterion unanimously indicated that the joint-density model was preferred (Table 1). To provide additional support for the joint-density model, we computed the correlation between the simulated and observed bias magnitudes as a function of the 18 discretized probe distances. First, we found that both the observed and simulated bias magnitudes increased as the physical probe distance increased (observed: *r* = .66, *p* < .002 for color; *r* = .55, *p* = .018 for shape; simulated: *r* = .94, *p* < .001 for color; *r* = .94, *p* < .001 for shape). More importantly, the simulated bias magnitudes predicted the observed bias magnitudes for both stimuli, even though the model was not fitted separately for each distance (*r* = .75, *p* < .001 for color; *r* = .45, *p* = .037 for shape; Fig. 6b).

**Table 1.** Model-Fit Comparisons for Experiments 1a and 1b

Experiment and model	∑ log-likelihood	AIC	BIC
Experiment 1a: shape			
Joint density (κ_P_)	**–179.7096**	**391.4191**	**482.6021**
Mixture density (a, κ_P_)	–294.6754	653.3508	835.7167
Experiment 1b: color			
Joint density (κ_P_)	**–301.0448**	**634.0897**	**723.0092**
Mixture density (a, κ_P_)	–476.1254	1,016.251	1,194.09

Note: Free parameters are given in parentheses (κ_P_ = precision of probe representation; a = mixture parameter). Boldface indicates the preferred model. AIC = Akaike information criterion; BIC = Bayesian information criterion.

## Experiment 3

We found convincing evidence that representational integration likely underlies the similarity-induced memory bias. One novel prediction of the joint-density model is that individuals with lower WM precision should exhibit a larger similarity-induced memory bias because of greater representational overlap between WM and probe representations (Fig. 5a; for a simulation, see the supplementary material available at https://osf.io/79kbm/). To test this, we examined the correlation between individuals’ WM precision and the magnitude of the similarity-induced memory bias using a variant of the paradigm employed in Experiment 1.

### Method

#### Participants

A power analysis based on the effect size obtained in Experiment 2 (*r* < –.32 between WM precision and the bias magnitude) determined that at least 99 samples would be necessary to detect such an effect with a statistical power of .9. The informed-consent procedure was the same as in previous experiments, and 124 individuals (97 female; mean age = 18.6 years) participated. The data from 14 participants were removed on the basis of the same exclusion criteria used in Experiment 2.

#### Procedure

The experiment was identical to Experiment 2 except for the following. There was one experimental condition with one intervening similarity judgment and one delay-matched baseline condition. The stimulus onset asynchrony between the memory item and the response wheel was set to 4,000 ms for both conditions. Participants performed four blocks consisting of 15 trials each of the baseline and experimental conditions (30 trials per block) in a pseudorandomized order.

#### Analysis

Memory precision was estimated by fitting the concentration parameter (κ) of a von Mises distribution to the response offsets for high-confidence reports in the baseline condition (> 69% of trials, or > 41 trials) to eliminate the effect of guessing. For the bias estimation, we focused on trials with high-confidence response offsets (> 62% of trials, or > 37 trials; see the supplementary material available at https://osf.io/79kbm/) for which participants successfully identified the similar probe. The precision estimates were then correlated with the bias estimates.

### Results

**Fig. 7.**
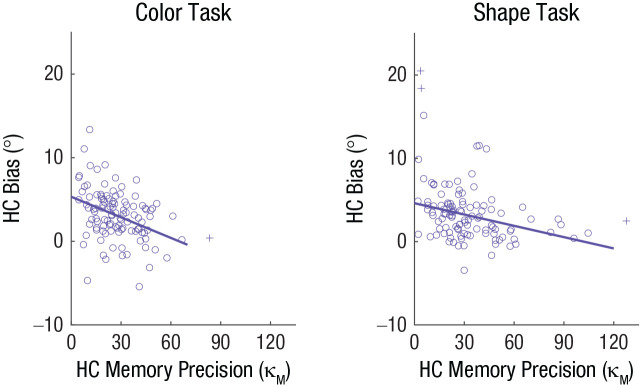
Results of the color task and shape task in Experiment 3. Each scatterplot depicts the relationship between bias for high-confidence (HC) reports and HC memory precision. Circles indicate individual participant data. Crosses indicate participant data excluded from the correlation as outliers. The solid lines indicate the best-fitting regressions.

Participants’ accuracy in the perceptual-comparison task was near ceiling (proportion of correct responses: *M* = .96, *SD* = .03 for the shape task; *M* = .97, *SD* = .03 for the color task), and we replicated the similarity-induced memory bias (see the supplementary material available at https://osf.io/79kbm/). More importantly, participants with high visual WM precision exhibited a smaller memory bias than those with low precision, *r*(107) = −.37, *p* < .001 for color; *r*(105) = −.31, *p* = .001 for shape (Fig. 7), as predicted by the joint-density model. This pattern was not changed when analyses were conducted with outliers that were initially excluded, *r*(108) = −.37, *p* < .001 for color; *r*(108) = −.33, *p* < .001 for shape.

## General Discussion

The present study revealed that the use of WM in perceptual comparisons results in a systematic memory bias, thus demonstrating that WM is susceptible to interference even without task-irrelevant disruptions of active maintenance. This memory bias was particularly large when a visual input was subjectively similar to the WM. Model comparisons showed that this similarity-induced memory bias was better characterized as representational integration between the WM and a subjectively similar input (i.e., joint-density model) than probabilistic confusion between the two (i.e., mixture-density model). We also provided empirical support of this representational-integration account by demonstrating that individuals with lower WM precision exhibited a larger memory bias than those with higher precision.

One may argue that the similarity-induced memory bias is solely determined by WM precision, thus questioning the causal role of perceptual comparisons in modulating the bias. More precisely, low-precision WM may be susceptible to greater stimulus-driven interference by a perceptual input, which may also result in perceived similarity between them. In other words, perceived similarity between WM and a perceptual input may be a mere by-product of low WM precision. The present study alone does not provide a strong test for this possibility because we did not measure WM precision prior to the perceptual comparison. However, we tested this possibility in a follow-up study (Saito et al., 2020) by having participants either ignore or compare intervening perceptual input with a WM representation in different experimental blocks. We found that identical intervening inputs induced a larger memory bias in *compare* blocks than in *ignore* blocks, and more importantly, we found that WM precision for the baseline condition (i.e., no intervening input) in both block types was statistically indistinguishable. These results demonstrated that the variability in WM precision alone could not explain the memory bias and, thus, implicated a causal role of similarity judgments in modulating the similarity-induced memory bias.

The temporal stability of the similarity-induced memory bias also merits further discussion. Wang and colleagues (2018) demonstrated that a WM representation can be distorted by similar perceptual inputs only when they appear before attention returns to the WM from a secondary task. Although we did not use any secondary task to distract attention away from the original WM representation prior to the onset of the similar probe, it is possible that the similarity-induced memory bias happens only when a WM is compared with a similar input too soon after encoding. We tested this possibility in a follow-up study (Saito et al., 2021) by directly manipulating the interval between memory encoding and similarity judgments. Here, we replicated the similarity-induced memory bias even when participants performed a similarity judgment between a perceptual input and a memory encoded 24 hr before by retrieving it into WM (Atkinson & Shiffrin, 1968; Cowan, 1999; Fukuda & Woodman, 2017). Furthermore, when participants retrieved a memory again 24 hr after a similarity judgment, the memory reports remained biased. This temporal stability of the similarity-induced memory bias is in stark contrast to the memory bias caused by a temporary disruption of active WM maintenance, thus suggesting that it is the direct usage of WM in perceptual comparisons that “locks in” the bias. Future studies will be necessary to determine the neural mechanisms that underlie these two distinct forms of memory biases.

Our representational-integration account of WM bias might offer a unifying explanation for other WM biases reported in the literature. For instance, past visual experiences can bias subsequently encoded WM—a phenomenon known as *serial dependence in visual perception* (Bae & Luck, 2019, 2020; Cicchini et al., 2018; Czoschke et al., 2019; C. Fischer et al., 2020; J. Fischer & Whitney, 2014; Kiyonaga et al., 2017). Although our finding that WM is biased by subsequently perceived inputs is different from serial dependence in the direction of causality, serial dependence might also be explained by the representational integration triggered by the subjective similarity between lingering WM representations of past visual experience and current WM representations.

Lastly, future studies should examine at what stage the similarity-induced memory bias occurs. One possibility is that the memory bias might be introduced to WM as soon as its similarity to a novel input is perceived. Alternatively, the WM might stay intact at the time of the perceptual comparison, and the bias might be introduced at the time of memory report. To tease apart these two hypotheses, future studies should incorporate neural-decoding approaches (Bae, 2021; Bae & Luck, 2018; Rademaker et al., 2019) to track the content of WM as it is maintained, compared with a perceptual input, and eventually reported.

## Transparency

*Action Editor:* Barbara Knowlton

*Editor:* Patricia J. Bauer

*Author Contributions*

K. Fukuda, A. E. Pereira, and H. Tsubomi designed the study. A. E. Pereira and J. M. Saito collected the data. K. Fukuda analyzed the data. G.-Y. Bae conducted the modeling analysis. All the authors wrote the manuscript and approved the final manuscript for submission.
